# Supplementary material for: L-Carnitine Tartrate Supplementation for 5 Weeks Improves Exercise Recovery in Men and Women: A Randomized, Double-Blind, Placebo-Controlled Trial
Source: Nutrients. 2021 Sep 28;13(10):3432. doi: 10.3390/nu13103432 (PMC8541253; doi:10.3390/nu13103432)
Supplement: Supplementary file 1 [file nutrients-13-03432-s001.zip › nutrients-1385660-supplementary.pdf]

Figure S1. Salivary Immunoglobulin A (sIgA).

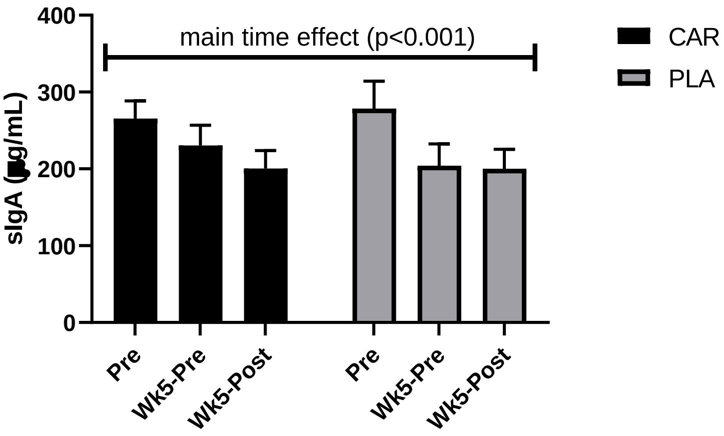

Figure S1: Bar chart for the mean values and error bars for the standard error of the mean for salivary IgA are shown for Pre, Wk5-Pre and Wk5-Post. The groups were carnitine (CAR) and placebo (PLA).

Table S1. Repetition Count.

	**CAR (n=37)**	**PLA (n=36)**
**D-Leg**	138 ± 12	127 ± 11
**ND-Leg**	125 ± 9	126 ± 11
**Total**	263 ± 19	253 ± 22

D = dominant, ND = non-dominant. Data is mean ± SEM.

Table S2. Body Composition.

	**CAR**		**PLA**	
	PRE	Wk5-Pre	PRE	Wk5-Pre
**Total Mass (kg)**	71.82±2.28	72.59±2.21	73.11±2.98	73.55±2.99
**Fat-Free Mass (kg)**	50.92±2.04	51.62±1.98	52.24±2.42	52.80±2.42
**Fat Mass (kg)**	20.91±0.96	20.97±0.96	20.87±0.83	20.75±0.85
**Body Fat (%)**	29.4±1.2	29.1±1.2	28.9±0.8	28.6±0.8

Data is mean ± SEM
